# Impact of Parenteral Copper and Zinc Administration on Reproduction, Inflammation, and Antioxidant Responses of *Bos indicus* Beef Heifers

**DOI:** 10.3390/ani15192926

**Published:** 2025-10-09

**Authors:** Luana Gomes da Silva, Marcelo Vedovatto, Juliana Ranches, Edilane Costa Martins, Matheus Fellipe Ferreira, Eduardo de Assis Lima, Luiz Carlos Louzada Ferreira, Willian Vaniel Alves dos Reis, Gumercindo Loriano Franco

**Affiliations:** 1 Faculdade de Medicina Veterinária e Zootecnia, Universidade Federal de Mato Grosso do Sul, Campo Grande 79070-900, MS, Brazil; luana@ciapecuaria.com.br (L.G.d.S.); assiseduvet@gmail.com (E.d.A.L.); willian.vet.ufms@gmail.com (W.V.A.d.R.); gumercindo.franco@ufms.br (G.L.F.); 2 Dean Lee Research and Extension Center, Louisiana State University, Alexandria, LA 71302, USA; 3 Eastern Oregon Agricultural Research Center, Oregon State University, Burns, OR 97720, USA; juliana.ranches@oregonstate.edu (J.R.); edilane.costamartins@oregonstate.edu (E.C.M.); 4 Hill Farm Research Station, Louisiana State University, Homer, LA 71040, USA; mferreira@agcenter.lsu.edu; 5 Cia Pecuária Assessoria, Campo Grande 79003-140, MS, Brazil; caio@ciapecuaria.com.br

**Keywords:** ceruloplasmin, glutathione peroxidase, haptoglobin, Nellore, superoxide dismutase

## Abstract

**Simple Summary:**

Copper (Cu) and zinc (Zn) are essential trace minerals for growth, reproduction, and immune function in cattle, but their supplementation is often challenging in grazing systems. We conducted two experiments to evaluate whether a single injection of a CuZn solution given before artificial insemination could improve the performance of beef heifers. In a small-scale experiment, heifers receiving CuZn tended to gain more weight and showed signs of enhanced antioxidant activity and ovarian function. In a larger experiment designed to test reproductive outcomes, heifers injected with CuZn were heavier, had greater blood Cu concentrations, and those with a low body condition score showed improved estrus activity and tended to have greater pregnancy rates. These findings suggest that CuZn injection may be a practical strategy to support the growth and reproductive success of beef heifers, particularly when their body condition is suboptimal.

**Abstract:**

Two experiments evaluated the effects of copper (Cu) and zinc (Zn) injection on body weight (BW), body condition score (BCS), pregnancy rate, ovarian traits, and antioxidant and inflammatory responses of beef heifers. In Exp. 1, 29 heifers were assigned to having saline or CuZn (a solution containing 15 and 50 mg/mL of Cu and Zn) subcutaneously injected (5 mL/heifer) 9 days before artificial insemination. Exp. 2 was conducted to increase the statistical power to evaluate pregnancy rate, and 283 heifers were assigned to either the saline or CuZn group. In Exp. 1, CuZn heifers tended (*p* ≤ 0.10) to gain more BW and to have greater corpus luteum size and plasma concentration of glutathione peroxidase. No effects of treatment were detected (*p* ≥ 0.18) for BCS; estrus score; serum concentration of Cu, Zn, and cortisol; and plasma concentration of haptoglobin, ceruloplasmin, and superoxide dismutase. In Exp. 2, CuZn heifers had greater (*p* < 0.01) BW and serum Cu. The CuZn heifers with low BCS had greater (*p* ≤ 0.05) estrus scores and tended (*p* = 0.10) to have greater pregnancy rates. Thus, injecting CuZn may be an effective strategy to enhance growth, reproductive performance, and antioxidant responses in heifers, especially when their BCS is below 5.

## 1. Introduction

Minerals play a vital role in animal metabolism, and their deficiency can lead to significant nutritional imbalances, resulting in suboptimal productive and reproductive performance [1,2]. To overcome issues with trace mineral (TM) deficiency, grazing cattle need to be frequently supplemented, as forages often fail to meet the cattle’s requirements [2,3]. A deficient or marginal TM status is often linked with impaired reproductive performance, such as reduced conception rates and increased incidence of anestrus, fetal resorption, placental retention rates, abortions, premature calving, cystic ovaries, and metritis [4].

The TM supplementation can be delivered through various methods, including free-choice oral mixes, salt blocks, drenches, rumen boluses, and injectable formulations. The parenteral supplementation has traditionally included vitamins A and E, along with TM such as copper (Cu), zinc (Zn), manganese (Mn), and selenium (Se), thus commonly referred to as injectable trace minerals (ITM). Studies evaluating the use of ITM have been associated with improved fertilization and pregnancy rates, as well as a reduced calving-to-conception interval [5,6]. The main described function of Cu and Zn in animal metabolism is to be part of the antioxidant enzyme called Cu-Zn superoxide dismutase, which is fundamental to controlling the oxidative stress that could impair reproduction [2]. Specifically, Cu deficiency is closely linked to impaired reproductive functions, highlighting the importance of Cu supplementation for successful pregnancy outcomes. Adequate Cu status is associated with normal reproductive functions, as it is linked to numerous enzymatic functions and antioxidant activity [2,7]. The Zn also plays a crucial role in reproductive success by directly influencing hormone secretions and function (e.g., progesterone, insulin, prostaglandin F2 alpha (PGF2α)), as well as enhancing oocyte and embryo viability, and supporting fetal development, thereby contributing to maintaining a viable pregnancy [8,9,10]. Furthermore, Zn modulates the secretion, bioavailability, and function of insulin growth factor-1 (IGF-1), a key mediator of reproductive processes including uterine involution, embryo implantation, and fetal growth [11].

Research has shown that strategic TM supplementation, particularly with a combination of traditional supplementation and ITM administration, can be an effective approach to enhancing reproductive performance in cattle [11,12,13,14]. Furthermore, the use of ITM, especially when animals are going through challenging events, such as calving, branding, breeding, weaning, and transportation, is a convenient tool to boost their mineral status quickly, thus avoiding problems associated with inconsistent intake of traditional mineral supplementation often observed during stressful and challenging periods [2,15]. It is essential to note that ITM should not replace traditional free-choice mineral supplementation. This technology should be utilized as a tool to enhance cattle mineral status before challenge events and/or as a complement to traditional oral supplementation strategies, particularly when these traditional strategies do not properly fit the production system [2]. During fixed-time artificial insemination (FTAI), cattle are brought to and held at working facilities multiple times during the scheduled protocol, potentially disrupting their routine behavior of free-choice mineral consumption, which justifies the use of ITM during this period.

Therefore, two experiments were conducted to evaluate the effects of a CuZn injection at the beginning of the FTAI protocol on growth, reproduction, inflammatory, and antioxidant responses of Nellore heifers.

## 2. Materials and Methods

The Institutional Animal Care and Use Committee (IACUC) of the Universidade Federal de Mato Grosso do Sul (UFMS) reviewed and approved the experimental protocols under protocol n° 754/2016. The experiments were conducted from October 2023 to March 2024.

### 2.1. Animals, Treatments, and Samples Collection

#### 2.1.1. Experiment 1

The experiment was conducted at the farm school of UFMS in Terenos, MS, Brazil (20°26′50.8″ S, 54°50′21.5″ W). A total of 29 Nellore heifers were enrolled in the experiment [body weight (BW) = 368 ± 54.1 kg; body condition score (BCS, i.e., a subjective scale to estimate the amount of fat in the heifers) = 4.80 ± 0.71]. Heifers were maintained in a single 12 ha pasture of Marandu grass [*Urochloa brizantha* (Hochst. ex A. Rich) R. D. Webster, cv. Marandu] and supplemented with a free-choice minerals and vitamins mix, with an expected supplement intake of 120 g/animal day (Table 1).

The experiment started 9 days before FTAI and ended 60 days after the insemination (d −9 to d 60). On d −9, heifers were stratified by BW and BCS and then randomly assigned to 1 of 2 treatments. (1) Saline (*n* = 15): injection of a saline solution (0.9% NaCl; 5 mL/heifer). (2) CuZn (*n* = 14): injection of a CuZn solution [Suplenut, Biogénesis Bagó, Curitiba, PR, Brazil; 15 and 50 mg/mL of Cu (as Cu edetate) and Zn (as Zn edetate), respectively; 5 mL/heifer; about 0.20 and 0.68 mg of Cu and Zn per kg of BW, respectively]. Solutions were applied subcutaneously on the right side of the neck of each animal. The day of application (nine days before the insemination) was defined based on the work of Vedovatto et al. [3], who observed that the peak of superoxide dismutase (SOD) happened about 8–12 days after the TMI, and our goal was to have increased plasma antioxidant enzyme concentration on the day of the insemination and later.

All heifers were assigned to an FTAI protocol from d −9 to 0. On d −9, heifers were administered a 2 mg intramuscular injection of estradiol benzoate (Gonadiol; Zoetis, São Paulo, Brazil), and an intravaginal progesterone-releasing insert containing 1.9 g of progesterone (P4; CIDR; Zoetis) was inserted. On d −2, the progesterone device was removed, and intramuscular injection of PGF2α (12.5 mg/heifer; Lutalyse; Zoetis), estradiol cypionate (0.6 mg/cow; ECP; Zoetis), and eCG (200 IU/cow; Novormon; Zoetis) were applied. On d 0, all heifers were inseminated by a single technician using semen from a single Nellore bull. On d 14, heifers were exposed to a single clean-up Nellore bull.

The BW and BCS (1–9 scale) were recorded on d −9 and d 60, according to Herd and Sprott [18], by a single evaluator. For evaluation of the estrus expression score, on d −2, all heifers were marked in the sacral region using a paint stick (RaidexMaxi; RAIDEX GmbH, Dettingen/Erms, Germany). Then, on d 0, the estrus expression score was evaluated according to the degree of paint removal, where 1—no paint removal = no estrus expression; 2—partial paint removal = low estrus expression; 3—complete paint removal = high estrus expression [19]. In addition, the mating rate was estimated based on the heifers’ estrus expression score, where heifers with an estrus expression score of 1 were classified as not mounted, and scores 2 and 3 as mounted [20]. The dominant follicle diameter (d 0), corpus luteum (CL) diameter (d 7 and 14), pregnancy status (d 60), and conceptus size (crown-rump and thoracic; d 60) were assessed using transrectal ultrasonography (7.5 MHz transducer; Mindray DP 2200 VET, Shenzhen, China). The CL volume (cm^3^) was calculated using the formula for the volume of the sphere [V = 4/3π(D/2)3], where D is the maximum diameter (mm) of the CL [21]. All technicians were blinded to treatments for all variables evaluated.

Blood samples were collected from a subsample of 10 heifers per treatment, from the coccygeal vein on d −9, 0, 7, 14, and 60 into two 10 mL blood collection tubes (Vacutainer, Becton Dickinson, Franklin Lakes, NJ, USA) with sodium heparin (for plasma collection) and without sodium heparin (for serum collection). Immediately after collection, blood samples were stored on ice and then centrifuged at 1200× *g* for 30 min to separate plasma and serum. Samples were stored at −20 °C for further plasma analysis of progesterone, haptoglobin, ceruloplasmin, SOD, glutathione peroxidase (GSH-Px), and serum concentrations of Cu, Zn, and cortisol. Progesterone was analyzed on d 7 and 14, and haptoglobin, ceruloplasmin, SOD, GSH-px, cortisol, Cu, and Zn on d −11, 0, and 60.

Forage samples were collected on d −9 and 60 using the grazing simulation [22]. Afterward, samples were dried in a ventilation oven at 55 °C for 5 d, ground in a 1 mm grain size sieve, and combined into a single composite sample for later chemical composition and mineral analysis.

#### 2.1.2. Experiment 2

The experiment was conducted in a commercial cow-calf operation (Fazenda Seriema) located in Miranda-MS, Brazil (20°24′02.0″ S, 56°18′11.2″ W). A total of 283 Nellore heifers [BW = 333 ± 31.1kg; BCS = 6.10 ± 0.80] were managed similarly and kept in 3 management groups due to grazing logistics [80, 94, and 109 heifers/group, respectively; pasture size of 80 ha]. Heifers grazed Marandu grass in a rotational grazing system and were supplemented with a free-choice minerals and vitamins mix with an expected supplement intake of 120 g/animal day (Table 1).

The experiment started 9 days before the AI and ended 120 days after the AI (d −9 to d 120). On d −9, within each group, heifers were randomly assigned to 1 of 2 treatments: saline (*n* = 140, i.e., 40, 42 and 58 heifers in groups 1, 2 and 3, respectively) or CuZn (*n* = 143, i.e., 40, 42 and 61 heifers in groups 1, 2 and 3, respectively), as described in Exp. 1. The FTAI protocol used was also similar to the one used in Exp. 1. The pregnancy rate was diagnosed by ultrasound (Mindray DP 2200 VET with 7.5 MHz transducer) on d 30, 70, and 120. Females that failed to become pregnant by d 30 were resynchronized in a new protocol, and females that failed to become pregnant on d 70 were exposed to bulls at a rate of 1/40. Bulls were removed on d 120 when the final pregnancy diagnosis was conducted. This management strategy, involving multiple rounds of FTAI, is commonly and routinely adopted by beef operations in Brazil. For evaluation of the estrus expression score, on d −2 (relative to the first FTAI) and 37 (relative to the second FTAI), heifers were marked in the sacral region using a paint stick (RaidexMaxi; RAIDEX GmbH, Dettingen/Erms, Germany). Then, on d 0 and 39, the estrus expression score and mating rate were evaluated as previously described for Exp. 1. The BW and BCS of heifers were evaluated on d −9, 30, 70, and 120 similarly to as previously described. All technicians were blinded to treatments for all variables evaluated.

Ten heifers per treatment from the same group were randomly selected to collect blood samples. Samples were collected from the coccygeal vein on d −9, 0, and 60 into two 10 mL blood collection tubes (Vacutainer, Becton Dickinson) with sodium heparin (for plasma collection) and without sodium heparin (for serum collection). Samples were processed and stored as was conducted in Exp. 1.

Forage samples were collected in 3 moments (d −9, 30, 120), as described in Exp. 1. Afterward, samples were dried in a ventilation oven at 55 °C for 5 d, ground to 1 mm, and combined into a single composite sample for chemical and mineral composition analysis.

### 2.2. Laboratory Analysis

Forage samples (Exp. 1 and 2) were analyzed according to (AOAC) [23] dry matter (DM), method 930.15; crude protein (CP), method 976.05; ether extract (EE), method 920.39; ash, method 942.05; and minerals (method 985.01). The concentrations of lignin, neutral detergent fiber (NDF), and acid (ADF) were analyzed as described by Van Soest et al. [24]. The total digestible nutrients (TDN) concentrations were calculated as described by Weiss et al. [17] and net energy for maintenance (NEm) and gain (NEg) by the equations proposed by the NASEM [16].

Plasma progesterone concentrations were determined using a solid-phase, competitive, chemiluminescent enzyme immunoassay (Immulite 1000, Diagnostics Products Corp., Los Angeles, CA, USA) previously validated for bovine samples [25]. The detectable range and intra-assay CV for plasma progesterone concentrations were 3.15 to 9.90 ng/mL and 1.32%, respectively. Serum concentrations of Cu and Zn were analyzed by a commercial laboratory (Axys Análises, Porto Alegre, RS, Brazil) as described by Bordignon et al. [26]. Plasma concentrations of haptoglobin were analyzed as described by Cooke and Arthington [27] and ceruloplasmin as described by Demetriou et al. [28]. The inter- and intra-assay CV was 3.9% and 9.4% for haptoglobin and 2.0% and 4.3% for ceruloplasmin, respectively. Samples were analyzed for cortisol (Immulite 1000; Siemens Medical Solutions Diagnostics, Los Angeles, CA, USA) as previously described [29] due to 100% cross-reactivity between bovine and human cortisol and accomplished within a single assay with an intra-assay CV of 8.52%. Plasma samples were also analyzed for SOD and GSH-Px using commercial ELISA kits (Cayman Chemical, Ann Arbor, MI, USA). The inter- and intra-assay CV was 4.3% and 5.7% for SOD, and 6.3 and 7.8% for GSH-px, respectively.

### 2.3. Statistical Analysis

Sample size calculations were performed using the POWER procedure of SAS (version 9.4; SAS Institute Inc., Cary, NC, USA). The number of animals per treatment was sufficient to detect at least 10% differences between treatments for all evaluated variables, assuming a *p*-value of 0.05 and 80% power based on a two-sided *t*-test. Estimates for sample size calculations were derived from prior experiments from our group [3,14].

For all analyses, the animal was considered the experimental unit. In Exp. 1, BCS and BCS change, ovarian variables, conceptus size, and plasma and serum variables were tested using the MIXED procedure of SAS with Satterthwaite approximation to determine the denominator degrees of freedom for the test of fixed effects. Data for BCS change, ovarian variables, plasma progesterone, and conceptus size were tested for the fixed effect of treatment, using animal(treatment) as a random effect, and BCS obtained on d −9 as a covariate. Data of BCS and serum and plasma variables were analyzed as repeated measures and tested for effects fixed of treatment, day, and resulting interaction, and for random effects of animal (treatment), and BCS obtained on d −9 was included as a covariate. In addition, the baseline data collected at d −9 of each variable were also included as covariates but removed from the model when *p* > 0.10. The covariance structures were selected based on the lowest values of the Akaike information criterion. Binary variables (e.g., pregnancy rates) were analyzed using the GLIMMIX procedure of SAS with the binomial distribution option and with Satterthwaite approximation to determine the degrees of freedom for tests of fixed effects. The model was tested for the fixed effect of treatment and random effects of cow (treatment), and the BCS obtained on d −9 was included as a covariate. In Exp. 2, the models used were like those described in Exp. 1, except for the random effect that included animal (treatment × management group) and management group in the model. In Exp. 2, post hoc analysis was also performed, where the cows were stratified by BCS on d −9 into two categories: low BCS (<5) and high BCS (≥5). The value 5 represented the median of BCS all animals in Exp. 2. Means were separated using the protected least significant difference (PDIFF; *t*-test), and all results were reported as least squares mean (LSMEANS) followed by the standard error of the mean (SEM). Significance was defined as *p* ≤ 0.05, and tendency when *p* > 0.05 and ≤0.10.

## 3. Results

### 3.1. Experiment 1

**Figure 1:**
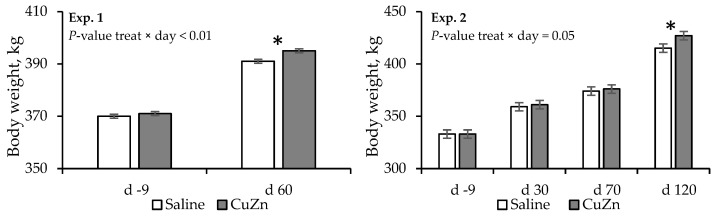
Body weight of heifers receiving a saline solution injection (saline) or a Cu and Zn solution injection (CuZn) at the beginning of fixed-time artificial insemination (d −9; Experiments 1 and 2). The symbol (*) highlights the days that significant differences were detected (*p* ≤ 0.10) between treatments.

There was a treatment × day interaction (*p* < 0.01) for BW (Figure 1), where heifers receiving CuZn treatment were heavier on d 60 than those receiving saline treatment. Moreover, a tendency (*p* = 0.09) in BW change was observed, where heifers receiving the CuZn treatment showed a greater BW change compared to those receiving the saline treatment. Effects of treatment × day and treatment were not detected (*p* ≥ 0.52) for BCS or BCS change (Table 2).

Effects of treatment were not detected (*p* ≥ 0.25) for estrus score or mating rate. Dominant follicle diameter and corpus luteum diameter on d 7 were not affected by treatment; however, corpus luteum diameter on d 14 tended (*p* = 0.10) to be greater in heifers receiving CuZn treatment compared to heifers receiving saline treatment (Table 2). Moreover, no effects of treatment were detected (*p* ≥ 0.15) for corpus luteum volume (d 7, d 14), plasma progesterone (d 14), or pregnancy rate (d 60). Regarding conceptus size traits, no effects of treatment on crown-rump (d 60) and thoracic (d 60) were detected (*p* ≥ 0.27; Table 2).

No effects of treatment or treatment × day were detected (*p* ≥ 0.18) for serum Cu, serum Zn, plasma haptoglobin, plasma ceruloplasmin, serum cortisol, and plasma SOD (Table 3). A treatment × day tendency (*p* = 0.10) was detected for the plasma GSH-px concentration of heifers assigned to CuZn treatment. On d 30, the plasma GSH-px concentration of heifers assigned to CuZn treatment tended (*p* = 0.10) to be greater than heifers assigned to saline treatment (Table 3).

### 3.2. Experiment 2

A treatment effect (*p* < 0.01) was observed for serum Cu, where heifers receiving CuZn treatment had a greater concentration of Cu compared to heifers assigned saline treatment (Table 3). On the other hand, no effects of treatment or treatment × day for serum Zn, plasma SOD, and plasma GSH-px were detected (*p* ≥ 0.28; Table 3).

Effects of treatment × day and treatment were detected (*p* < 0.01) for BW (Figure 1). On d 120, heifers receiving CuZn treatment had greater BW compared to heifers receiving saline treatment. Moreover, a tendency (*p* = 0.09) in body weight change was observed from d −9 to d 30 and from d −9 to d 120, where heifers assigned to CuZn treatment tended to gain more weight than heifers assigned to saline treatment. There were no treatment or treatment × day effects (*p* ≥ 0.34) for BCS, or BCS change (*p* ≥ 0.22).

No effects were observed for the overall estrus expression score (*p* ≥ 0.16). However, when classifying heifers according to the BCS (BCS < 5 as Low Vs. BCS ≥ 5 as high), a treatment effect was detected (*p* = 0.01) for estrus expression score on d 0, where heifers classified as having low BCS had greater estrus expression score. No treatment effect was detected (*p* ≥ 0.16) in heifers with high BCS (Table 4). A treatment tendency (*p* = 0.10) for greater mating rate for heifers receiving CuZn treatment was observed on d 0. Furthermore, an effect of treatment (*p* = 0.04) was observed for mating rate on d 0 for heifers with low BCS, where heifers with low BCS receiving CuZn treatment presented a greater percentage of mating rate compared to heifers receiving saline treatment. Effects of treatment were not detected (*p* = 0.72) for mating rate of heifers with high BCS. No effects of treatment nor time (*p* ≥ 0.23) were observed for mating rate on d 39 (Table 4). Relative to pregnancy rates, heifers with low BCS receiving CuZn had a tendency (*p* = 0.10) to have a greater pregnancy rate on d 30 when compared to heifers treated with saline solution. No effect was observed (*p* ≥ 0.26) for heifers with high BCS, and overall, on d 30. No treatment effects were detected (*p* ≥ 0.25) for pregnancy rate on d 70 (2nd FTAI), d 120 (bull), and d 120 (FTAIs + bull) for heifers with low, high BCS, and overall. Final pregnancy loss determined on d120 was not affected by the treatments (*p* ≥ 0.16; Table 4).

## 4. Discussion

The effects of ITM administration reported in the literature on BW and BCS have been inconsistent [3,14,15,30]. Heifers receiving injectable supplementation of CuZn, in the current study, exhibited increased BW throughout the study and tended to have greater BW change compared to heifers receiving a saline injection. In contrast, Vedovatto et al. [3], working with Nellore cows, and Hernandez et al. [15], working with Angus × Hereford cows, and using ITM injections throughout the production cycle reported no effects of ITM administration on BW or BCS of cows, attributing the lack of growth response to adequate mineral and nutritional status of those herds. It is possible that in the current study, heifers might have been subjected to nutritional restrictions, and therefore ITM administration might have been beneficial. The improvement in BW observed in CuZn-injected heifers is a relevant finding, given that the majority of cattle worldwide are raised in regions where nutritional restrictions are common.

In the current study, no effect of ITM administration was observed on BCS. However, Vedovatto et al. [14] observed in their study that cows treated with ITM tended to have a greater BCS and gained BCS from d −11 to 30, whereas saline-treated cows had a decrease in BCS and had a lesser BCS during the same period. The authors reported that, because the cows had already reached maturity when the trial started, the effect of ITM observed may have occurred in the form of greater adipose tissue deposition. Additionally, Mundell et al. [31], reported a greater BCS increase in cows administered ITM 30 d before AI than in cows administered a saline injection. Although heifers in the current study receiving CuZn during FTAI gained BW, the change was insufficient to support changes in the BCS scale. According to the NRC [32], a body weight gain of approximately 30 kg would be required to promote a one-point change in BCS, while in this study, heifers gained approximately 25 kg.

There are contrasting reports on the effects of mineral supplementation on reproductive performance, and numerous factors can influence this inconsistency, such as reproductive management, breed, category, BCS, TM status at the time of mineral injection, etc. [7,33]. In this study, the CL size, which is positively correlated with plasma progesterone concentration Kastelic et al. [34] tended to be greater on d 14 for heifers receiving CuZn treatment compared to heifers receiving saline treatment, despite no observed difference in plasma progesterone (d 14) concentration. Similarly, Anchordoquy et al. [35] reported an increase in CL size in Zn-deficient cows administered an injection of Zn; however, in our previous experiment, we reported (Vedovatto et al. [14]) that the administration of ITM resulted in a reduced CL size. Finally, Gonzalez-Maldonado et al. [36] observed no effect of ITM supplementation to cows 30 d before AI on follicle population, dominant follicle size, time of estrus after CIDR removal, and CL size.

Heifers with low BCS on d 30 (1st FTAI) receiving CuZn treatment tended to have a greater pregnancy rate. Similarly, Vedovatto et al. [3] and Vedovatto et al. [14] reported a tendency to increase pregnancy rate only when cows treated with ITM had low BCS. Anchordoquy et al. [35] observed an increased pregnancy rate when cows were administered a Zn injection at the beginning of the FTAI protocol. Meanwhile, Arthington et al. [37] reported no effects of ITM on pregnancy rates and Springman et al. [38] reported no effects of ITM on the pregnancy rates of heifers when mineral status was adequate. The exact reasons for such responses are not very clear; however, Vedovatto et al. [14] suggested that this response might be attributed to the greater BCS for ITM cows in their study. In our current experiment, CuZn-treated heifers gained more weight, which may be associated with the higher reproduction performance observed. Alternatively, a better antioxidant status and increased mineral status, as observed in the current study, with greater GSH-px on d 30 and greater serum Cu concentration for heifers treated with CuZn, could also play a role in improving pregnancy rates. Greater antioxidant enzyme activity may have enhanced the capacity to mitigate oxidative stress and the associated risk of cellular damage [27]. The minerals Cu and Zn are components of the enzymes Cu-Zn SOD, and when TMI is applied to cattle, these enzymes are frequently increased [3,14]. The reason why the CuZn injection did not increase SOD concentration in the current experiment is unknown and deserves further investigation.

In our experiment, the CuZn injection did not affect the plasma concentration of haptoglobin and ceruloplasmin. The effects of TM injection on the acute phase response are contradictory in the literature. In a study conducted by Arthington et al. [37], the TM injection increased the plasma concentrations of haptoglobin by 6–10 d after the injection (1 mL/45kg of BW) in Brangus crossbred heifers, indicating a possible inflammatory reaction. Based on that experiment, Caramalac et al. [39] evaluated the inflammatory reaction in Angus heifers at weaning in response to TM (1 mL per 92 kg of BW), and no effects on haptoglobin or ceruloplasmin were detected. Furthermore, in two experiments conducted by our group, Vedovatto et al. [3,14], the TM injection (~1mL/66 kg of BW) before the FTAI protocol in Nellore beef cows also did not affect the plasma concentration of ceruloplasmin and haptoglobin. In the current experiment, the dosage used (~1 mL/74 kg of BW in Exp. 1) was lower than that used by Arthington et al. [37], which could explain why that experiment elicited inflammatory reactions but not our other studies.

Collectively improved BW, mineral, and antioxidant status seems to positively impact the reproduction efficiency of Nellore heifers. However, studies exploring the mechanisms by which ITM may increase the reproductive efficiency of cattle are warranted.

## 5. Conclusions

In summary, the use of injectable CuZn at the beginning of the FTAI protocol for Nellore heifers resulted in increased BW, estrus expression score, and mating rate, especially for heifers with a lower BCS, leading to a tendency for increased pregnancy rate, accompanied by greater serum Cu, and plasma GSH-px activity. The administration of injectable CuZn may be a viable strategy to complement conventional mineral supplementation and improve growth, reproduction, and antioxidant responses in Nellore heifers.

## Figures and Tables

**Table 1:** Chemical and mineral composition of forages and guarantee levels of free-choice minerals and vitamins supplement (Exp. 1 and 2).

Items	Exp. 1 (Farm School)	Exp. 2 (Commercial Operation)	Requirement ^1^
Forage			
g/kg of DM			
Crude protein	91.8	75.2	
Neutral detergent fiber	643	716	
Acid detergent fiber	349	393	
Lignin	57.2	70.0	
Ether extract	14.4	12.5	
Ash	102	86.3	
Calcium	1.98	1.77	
Phosphorus	1.93	1.78	
Sodium	0.37	0.77	1.0
Potassium	25.9	22.5	7.0
Magnesium	2.28	2.02	2.0
Sulfur	0.9	1.00	1.5
TDN ^2^	572	546	
Mcal/kg of DM			
NEm ^3^	1.21	1.13	
NEg ^3^	0.65	0.57	
mg/kg of DM			
Iron	431	109	50.0
Manganese	158	91.8	40.0
Selenium	0.11	0.12	0.10
Zinc	26.9	20.7	30.0
Copper	4.86	4.16	10.0
Mineral/vitamin supplement ^4^			
g/kg of DM			
Calcium	196	140–190	
Phosphorus	90	80	
Sodium	99	110	
Magnesium	20	10	
Sulfur	20	16	
mg/kg of DM			
Fluorine	900	880	
Cobalt	200	61	
Iodine	180	55	
Iron	2400		
Manganese	1670	4682	
Selenium	40	11.8	
Zinc	3000	3273	
Copper	1200	1091	
IU/kg of DM			
Vitamin A	150,000		
Vitamin D3	30,000		
Vitamin E	1500		
Target intake, g/d	100	200	

^1^ Requirements established by NASEM [16]. ^2^ Calculated as described by Weiss et al. [17]. ^3^ Calculated using the equations proposed by the NASEM [16]. ^4^ Guarantee levels; in Exp. 1 the mineral supplement used was Mega Fós 90 Milk (AgroMega Indústria de Alimentos Animal, Tamboara, PR, Brazil) and in Exp. 2, it was Probeef 800 (Nutron Nutrição Animal, Itapira, SP, Brazil).

**Table 2:** Body and reproductive variables of heifers receiving a saline solution injection (saline) or a Cu and Zn solution injection (CuZn) at the beginning of fixed-time artificial insemination (d −9; Experiment 1).

Items	Treatments ^1^		SEM	*p*-Value	
	Saline	CuZn		Trt	Trt × Day
Exp. 1, *n* =	15	14			
Body traits					
BW change, kg	20.5	24.4	1.51	0.09	
Body condition score (BCS), 1–9				0.52	0.63
d −9	4.81	4.90	0.14		
d 60	4.81	4.78	0.14		
BCS change, 1–9	0.00	−0.15	0.17	0.52	
Estrus traits					
Estrus expression score (d 0), 1–3	1.80	1.42	0.23	0.25	
Mating rate (d 0), %	75.3	76.4	0.12	0.95	
Ovarian traits					
Dominant follicle diameter (d 0), mm	10.7	10.4	0.87	0.77	
Corpus luteum diameter, mm					
d 7	18.5	19.2	0.93	0.59	
d 14	17.3	19.4	0.81	0.10	
Corpus luteum volume, cm^3^					
d 7	3.55	3.99	0.55	0.60	
d 14	2.99	4.00	0.44	0.15	
Plasma progesterone (d 14), ng/mL	5.68	6.42	0.67	0.44	
Pregnancy rate (d 60), %	40.8	45.2	0.14	0.84	
Conceptus size traits					
Crown-Rump (d 60), mm	32.9	30.6	2.14	0.51	
Thoracic (d 60), mm	15.3	13.2	1.12	0.27	

^1^ Treatments were saline solution (0.9% of NaCl) or CuZn solution [Suplenut, Biogénesis Bagó, Curitiba, PR, Brazil; 15 and 50 mg/mL of Cu (as Cu edetate) and Zn (as Zn edetate), respectively]. Both solutions were applied subcutaneously at a dose of 5 mL/heifer on the right side of the neck of each animal.

**Table 3:** Serum and plasma variables of heifers receiving a saline solution injection (saline) or a Cu and Zn solution injection (CuZn) at the beginning of fixed-time artificial insemination (d −9; Experiment 1 and 2).

Items ^1^	Treatments ^2^		SEM	*p*-Value	
	Saline	CuZn		Trt	Trt × Day
Exp. 1, *n* =	10	10			
Serum Cu, μg/dL	52.1	49.2	2.60	0.47	0.61
Serum Zn, μg/dL	51.9	48.1	4.60	0.58	0.91
Serum cortisol, µg/dL	3.05	3.52	0.24	0.18	0.73
Plasma haptoglobin, mg/mL	0.40	0.41	0.02	0.69	0.25
Plasma ceruloplasmin, mg/mL	16.4	16.6	0.54	0.75	0.97
Plasma SOD, U/mL	86.8	69.3	13.9	0.50	0.21
Plasma GSH-px, U/mL				0.12	0.10
d −9	71.5	71.6	7.57		
d 30	68.7 ^b^	94.4 ^a^	7.57		
d 60	67.6	79.7	7.57		
Exp. 2, *n* =	10	10			
Serum Cu, μg/dL	49.0	69.9	3.79	<0.01	0.28
Serum Zn, μg/dL	59.8	67.8	7.72	0.56	0.17
Plasma SOD, U/mL	88.0	83.3	12.0	0.78	0.96
Plasma GSH-px, U/mL	70.5	74.7	4.99	0.57	0.79

^1^ Superoxide dismutase (SOD) and glutathione peroxidase (GSH-px). ^2^ Treatments were saline solution (0.9% of NaCl) or CuZn solution [Suplenut, Biogénesis Bagó, Curitiba, PR, Brazil; 15 and 50 mg/mL of Cu (as Cu edetate) and Zn (as Zn edetate), respectively]. Both solutions were applied subcutaneously at a dose of 5 mL/heifer on the right side of the neck of each animal. ^a, b^ within a row means without a common superscript tend to differ (*p* ≤ 0.10).

**Table 4:** Body and reproductive variables of heifers receiving a saline solution injection (saline) or a Cu and Zn solution injection (CuZn) at the beginning of fixed-time artificial insemination (d −9; Experiment 2).

Items ^1^	Treatments ^2^		SEM	*p*-Value	
	Saline	CuZn		Trt	Trt × Day
Exp. 2, *n* =	140	143			
Body traits					
BW change, kg					
d −9 to 30	24.3	26.8	8.98	0.09	
d 30 to 70	13.6	14.4	5.01	0.61	
d 70 to 120	42.8	47.5	26.7	0.57	
d −9 to 120	76.9	87.8	27.8	0.09	
Body condition score (BCS), 1–9				0.34	0.44
d −9	5.56	5.58	0.05		
d 30	6.07	6.10	0.05		
d 70	5.94	5.92	0.07		
d 120	6.53	6.70	0.07		
BCS change, 1–9					
d −9 to 30	0.51	0.52	0.09	0.60	
d 30 to 70	−0.14	−0.18	0.18	0.81	
d 70 to 120	0.59	0.78	0.18	0.55	
d −9 to 120	0.97	1.12	0.09	0.22	
Estrus traits					
Estrus expression score, 1–3					
d 0					
Low BCS	2.59	2.82	0.07	0.01	
High BCS	2.67	2.62	0.09	0.68	
Overall	2.62	2.73	0.05	0.16	
d 39					
Low BCS	2.82	2.86	0.07	0.71	
High BCS	2.83	2.84	0.07	0.93	
Overall	2.82	2.85	0.05	0.69	
Mating rate, %					
d 0					
Low BCS	88.3	97.1	3.14	0.04	
High BCS	87.8	89.9	5.07	0.72	
Overall	87.8	93.7	2.58	0.10	
d 39					
Low BCS	100	96.0	0.02	0.23	
High BCS	100	100	-	-	
Overall	100	98.0	1.24	0.25	
Pregnancy rate, %					
d 30 (1st FTAI)					
Low BCS	47.9	56.1	5.10	0.10	
High BCS	48.0	52.8	6.88	0.61	
Overall	48.3	55.4	4.63	0.26	
d 70 (2nd FTAI)					
Low BCS	38.2	28.6	9.97	0.42	
High BCS	33.1	48.9	9.65	0.25	
Overall	36.2	38.7	7.26	0.79	
d 120 (bull)					
Low BCS	12.4	9.45	3.90	0.58	
High BCS	15.0	11.4	4.71	0.59	
Overall	13.6	10.2	2.92	0.43	
d 120 (FTAIs + bull)					
Low BCS	82.3	77.3	5.44	0.40	
High BCS	80.6	87.4	6.47	0.35	
Overall	81.7	82.1	4.12	0.94	
Pregnancy loss, %					
d 30 to 120					
Low BCS	0.00	2.50	1.81	0.33	
High BCS	3.66	3.05	3.48	0.90	
Overall	1.50	2.77	1.79	0.61	
d 70 to 120					
Low BCS	7.15	1.92	8.70	0.39	
High BCS	0.43	12.3	10.8	0.33	
Overall	3.65	16.5	6.30	0.16	

^1^ Heifers with BCS < 5 at the beginning of the fixed-time artificial insemination (FTAI) protocol were classified as “Low BCS” and BCS ≥ 5 as “High BCS”. ^2^ Treatments were saline solution (0.9% of NaCl) or CuZn solution [Suplenut, Biogénesis Bagó, Curitiba, PR, Brazil; 15 and 50 mg/mL of Cu (as Cu edetate) and Zn (as Zn edetate), respectively]. Both solutions were applied subcutaneously at a dose of 5 mL/heifer on the right side of the neck of each animal.

## Data Availability

Data are available by email upon request to the corresponding author with reasonable justification.
